# The intimate relationship between structural relaxation and the energy landscape of monatomic liquid metals

**DOI:** 10.1038/s41598-021-91062-0

**Published:** 2021-06-03

**Authors:** Franz Demmel, Louis Hennet, Noel Jakse

**Affiliations:** 1 ISIS Facility, Rutherford Appleton Laboratory, Didcot, OX11 0QX UK; 2 ICMN, CNRS and University of Orleans, 45071 Orléans, France; 3 University of Grenoble Alpes, CNRS, Grenoble INP, SIMaP, 38000 Grenoble, France

**Keywords:** Condensed-matter physics, Structure of solids and liquids

## Abstract

The characteristic property of a liquid, discriminating it from a solid, is its fluidity, which can be expressed by a velocity field. The reaction of the velocity field on forces is enshrined in the transport parameter viscosity. In contrast, a solid reacts to forces elastically through a displacement field, the particles are trapped in their potential minimum. The flow in a liquid needs enough thermal energy to overcome the changing potential barriers, which is supported through a continuous rearrangement of surrounding particles. Cooling a liquid will decrease the fluidity of a particle and the mobility of the neighbouring particles, resulting in an increase of the viscosity until the system comes to an arrest. This process with a concomitant slowing down of collective particle rearrangements might already start deep inside the liquid state. The idea of the potential energy landscape provides an attractive picture for these dramatic changes. However, despite the appealing idea there is a scarcity of quantitative assessments, in particular, when it comes to experimental studies. Here we present results on a monatomic liquid metal through a combination of *ab initio* molecular dynamics, neutron spectroscopy and inelastic x-ray scattering. We investigated the collective dynamics of liquid aluminium to reveal the changes in dynamics when the high temperature liquid is cooled towards solidification. The results demonstrate the main signatures of the energy landscape picture, a reduction in the internal atomic structural energy, a transition to a stretched relaxation process and a deviation from the high-temperature Arrhenius behavior of the relaxation time. All changes occur in the same temperature range at about $1.4 \cdot T_{melting}$, which can be regarded as the temperature when the liquid aluminium enters the landscape influenced phase and enters a more viscous liquid state towards solidification. The similarity in dynamics with other monatomic liquid metals suggests a universal dynamic crossover above the melting point.

## Introduction

The liquid state is limited by first order transitions to a crystalline state or at high temperature to the gas. If crystallization can be avoided the liquid can be undercooled and eventually the material morphs into a glass upon cooling. From structural point of view the liquid and glass state do not differ very much^1^, yet the dynamics changes dramatically towards the glass transition. The main characteristic property of a dense liquid, the ability to flow, is limited by the forces of the surrounding particles, captured by the thermodynamic transport parameter viscosity. Viscosity increases by many orders of magnitude in a small temperature range towards the glassy state^2^. That strong increase can be understood as a feedback mechanism when through cooling the surrounding particles become less mobile and hence hinder the flow of the tagged particle more and more through their interaction forces. Formally, mode coupling theory (MCT) was quite successful to describe the feedback mechanism in dynamics with the prediction of a critical temperature $T_C$, when structural relaxation stops^3^. The divergent increase of the viscosity is accompanied by a dramatic slowing down of the structural relaxation dynamics, characteristic for the decay of density fluctuations and hence for the mobility and fluidity on a microscopic length scale. A lot of effort has been devoted to establish the changes in dynamics towards the glass transition in the past^4^. Less simple to answer is the question at which temperature the slowing down sets in or whether such a temperature range exists at all. Quite some time ago it was postulated that a transition temperature exists, usually above the melting point, which signals emerging correlated length scales^5^. Later, for metallic liquids a scaling behavior for the macroscopic viscosity was reported which links the glass transition temperature with the departure temperature from an Arrhenius behavior^6^. For this transition temperature $T_A$ a value of about twice $T_g$ was derived. A further slow structural relaxation process has been identified above the melting point in a molecular dynamics simulation of a simple liquid metal and described by a mode coupling calculation^7^. It was suggested that this slow process is responsible for the freezing of the liquid and was later confirmed experimentally^8,9^. Further MD-simulations on binary alloys found changes in the dynamics far above the glass transition temperature and sometimes even far above the liquidus, which were related to dynamic heterogeneity and the onset of collective particle movements^10–14^.

A different viewpoint to the changes towards the glass transition is offered by the potential energy landscape picture^15,16^. Classical MD-simulations on a binary Lennard-Jones liquid delivered a change in the inherent potential energy more than a factor two above the glass transition temperature. It evoked the idea that with decreasing temperature the atoms will become more and more trapped within their potential basins and will be less mobile due to thermal energy activation. That temperature range when the landscape influenced dynamics sets in with cooling can be regarded as the crossover to solidification on the microscopic level. However, there do not exist many studies which combine directly the energy landscape picture with the structural relaxation dynamics changes, in particular, when it comes to support from experiment.

Here we present an experimental and computational study on a monatomic liquid metal, which demonstrates the onset of the landscape influenced atomic mobility within the internal potential energy at a temperature of about 350 K above the melting point. Concomitantly in the same temperature range the structural relaxation process slows down and the dynamics demonstrate the hallmark of entering the landscape influenced regime.

Liquid aluminium dynamics has been studied intensively before through classical, see for example^17–19^, and first-principles molecular dynamics simulations^20–26^. Experimentally, less studies exist with a focus on the collective inelastic dynamics^27^ and on the diffusion^28,29^. A temperature dependent study on the dynamics at next neighbor distances revealed a change to a more solid-like behavior about 350 K above the melting point^30^. Previously an investigation on the thermodynamics of solid and liquid aluminium was conducted, which concluded that upon heating the crystal has a high stability limit temperature around 350 K above the melting point^31^.

## Results

To reveal the structural relaxation dynamics the experiments focused on the dynamics at the structure factor maximum of liquid aluminium, which occurs at $Q_0=2.65$﻿Å^﻿-1^. At this wave vector density fluctuations on a next-neighbor distance are monitored and hence provide direct insight into the microscopic collective dynamics on atomic length scales.

**Figure 1:**
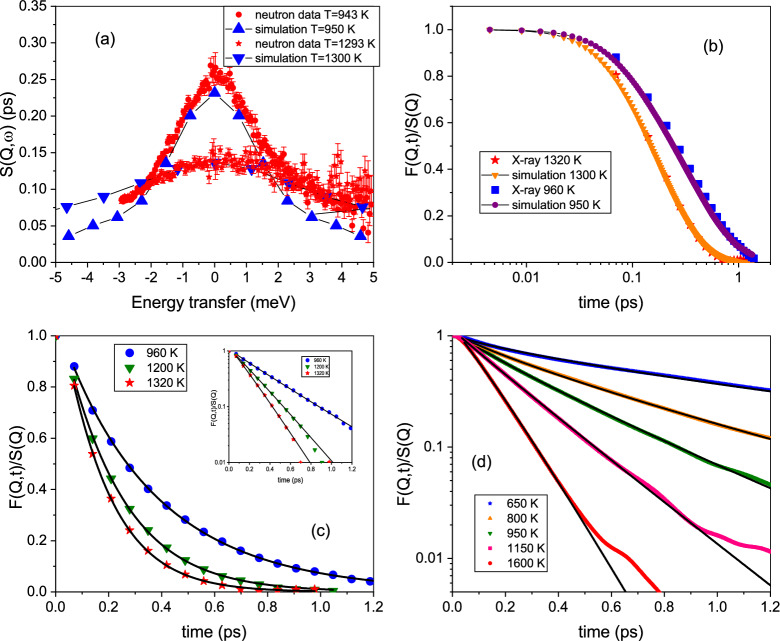
Energy and time spectra are presented, obtained through the different methods. Panel (**a**) shows a comparison between the neutron spectra and the AIMD spectra for two temperatures to demonstrate the nearly perfect agreement on lineshape and also absolute amplitude. Panel (**b**) displays a comparison between the intermediate scattering function $F(Q_0,t)$ from the Fourier transformed x-ray spectra and the simulation results for two temperatures on a logarithmic time axis. Panel (**c**) depicts experimental $F(Q_0,t)$ for three temperatures with KWW-function fits included as a line. The inset shows the data on a logarithmic scale to demonstrate the nearly exponential decay in this temperature range. Panel (**d**) shows the simulated $F(Q_0,t)$ data for 5 temperatures on a logarithmic scale including fits with KWW relaxation functions.

Figure 1 shows in four panels results from the scattering experiments in comparison with results from the AIMD simulation. Panel (a) depicts a direct comparison of $S(Q_0,\omega =0)$ of neutron and simulation spectra, demonstrating a remarkable good agreement on an absolute scale for the amplitude and lineshape. Panel (b) compares the intermediate scattering function $F(Q_0,t)$ from the simulation with the Fourier transformed function from inelastic X-ray scattering against a logarithmic time scale. Both comparisons demonstrate that the AIMD calculates the dynamic properties of liquid aluminium quite accurate. Panel (c) shows the intermediate scattering function from inelastic X-ray scattering and panel (d) $F(Q_0,t)$ from the simulation on a logarithmic scale for different temperatures. Included are fits as a line with a stretched exponential decay function, the Kohlrausch–Williams–Watt (KWW) function: $F(Q,t)/S(Q)=exp(-(t/\tau )^{\beta })$. A stretching parameter $\beta < 1$ describes a deviation of a simple exponential decay towards a more stretched relaxation in time. For very short times the particle experiences free particle dynamics with a Gaussian for the intermediate scattering function. As a limit for the ballistic particle propagation the inverse Einstein frequency, as an average collision or interaction frequency, can be assumed^32^. That estimate agrees well with previous simulation results which demonstrated a ballistic movement in liquid aluminium up to about $t \approx 0.03~ps$^24^ and sets the lower time limit for the relaxation dynamics fit.

**Figure 2:**
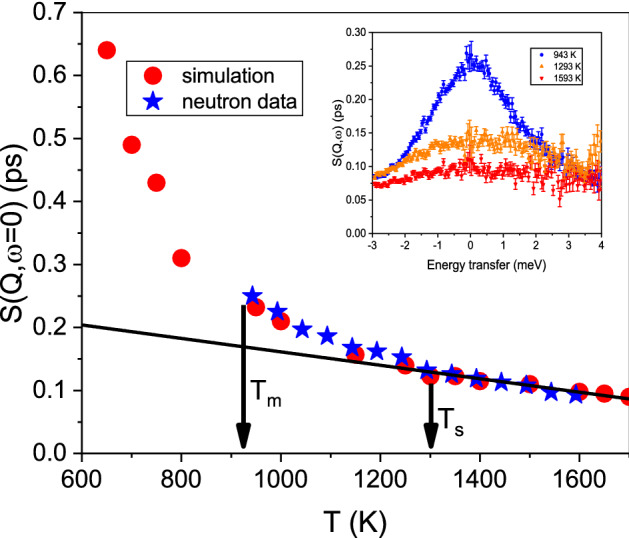
The amplitude $S(Q_0,\omega =0)$ from the neutron data and the simulation is plotted and demonstrates a good quantitative agreement. The inset shows three neutron spectra, which demonstrate that the amplitude changes between 943 and 1293 K around 3 times faster than for the higher temperature range. The line is a fit through the high temperature points as a guide for the eye, and evidences a slope change around 1300 K with a stronger increase in the amplitude $S(Q,\omega =0)$. Arrows indicate the melting temperature $T_m$, and the stability limit of solid Al (see text).

Figure 2 depicts the amplitude values $S(Q_0,\omega =0)$ from the neutron data and from the simulation. Included is a linear fit through the high temperature data points which indicates a changing slope around 1300 K, well above the melting temperature $T_m$. Interestingly, this change is close to a temperature $T_S=1292~K$, which was derived as a thermodynamic stability limit of the solid crystalline state of aluminium^31^, and beyond which no superheated metastable solid aluminium may survive.. That temperature agrees well with the increase in the amplitude $S(Q_0,\omega =0)$, which is a measure for the decay of density fluctuations on next-neighbor distances. The amplitude can be related directly to an average relaxation time for the density fluctuations^33^ and hence evidences a slowing down of the dynamics.

**Figure 3:**
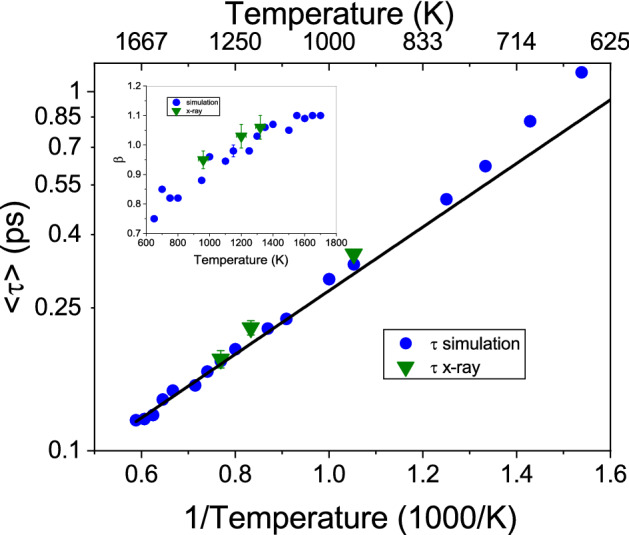
The relaxation times $<\tau>$ from the KWW-fits to the simulation and X-ray data are plotted against the inverse temperature on a logarithmic scale. The inset shows the resulting stretching parameter $\beta$. The line is a linear fit through the high temperature points ($T > 1400~K$) in this Arrhenius-type plot.

The intermediate scattering functions from the simulation and from the inelastic X-ray scattering experiment have been fitted with the KWW-function, neglecting the short time ballistic time range. The resulting relaxation times $\tau$ have been averaged into a stretch parameter free form according to: $<\tau >=\tau /\beta \Gamma (1/\beta )$ with the $\Gamma$-function. Figure 3 depicts the resulting relaxation times on a logarithmic scale against the inverse temperature in an Arrhenius-type plot. Note that the here presented structural relaxation time is similar but not identical to the Maxwell relaxation time, which characterizes the relaxation of shear deformations and shows a slightly different temperature dependence^34^. A line has been fitted through the high temperature values. The relaxation times deviate to slower times from this Arrhenius-type extrapolation and indicate a change towards slower dynamics around 1300 K. The inset depicts the stretching parameters $\beta$ from the fits, which increase with temperature and seem to saturate slightly above $\beta \approx 1$ at high temperature. The transition from a simple high-temperature exponential decay to a stretched dynamics is one of the signatures of the potential energy landscape predictions^15^. The slowing down of structural relaxation dynamics in liquid aluminium sets in about 350 K above the melting point.

**Figure 4:**
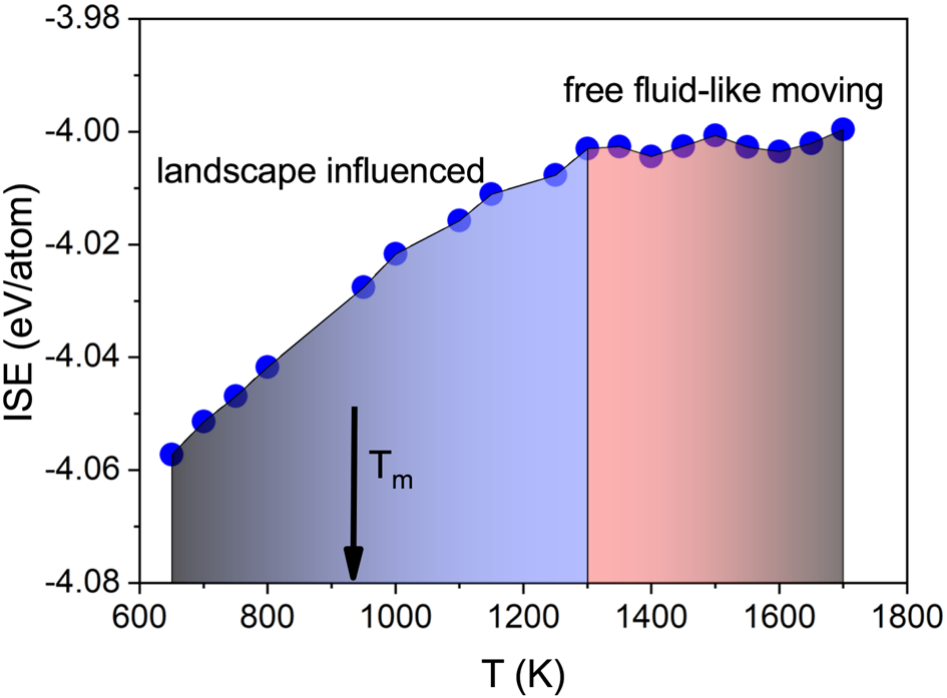
The inherent structure energy of Al is plotted against the temperature. Indicated are the two main regions of particle mobility according to the ISE within the energy landscape picture.

Figure 4 displays the averaged per atom inherent structure energy (ISE) against the temperature. Two different regions can be identified. Above 1300 K the ISE is practically constant. However, with lowering the temperature below 1300 K the ISE decreases continuously. According to the potential energy landscape picture the lowering of the ISE corresponds to the landscape-influenced regime, where atoms experience deeper basins from which they can not escape so easily anymore through thermal energy^2^. In contrast, at high temperature the thermal energy of the particles can compensate for the depth of the potential basins and the particles flow nearly freely.

## Discussion

The calculated averaged inherent structure energy shows above 1300 K a plateau-like behavior as a function of temperature. Below that temperature it continuously decreases and evidences a changing potential environment with increasing density.

The change in internal potential with temperature has direct consequences on particle dynamics and on the activation energies involved. As a matter of fact, the excess entropy shown in Fig. 6 of the supplement information is representative of the number of accessible states in the liquid^35,36^. The latter crossovers towards a steeper decrease below $T_S$, upon entering the landscape influenced regime, and as mentioned above, atoms become more and more trapped in energy barriers of the local minima^2^, leading to a decrease of the number of accessible states and hence slowing down the dynamics. The amplitude $S(Q_0,\omega =0)$ shows a changing slope around 1300 K with a much more strongly increasing amplitude with decreasing temperature. The amplitude is directly related to an average relaxation time, which shows a perfect agreement with simulation relaxation times. These averaged relaxation times evidence a non-Arrhenius behavior only below the melting point (see Suppl. Information). However, our detailed investigation of the relaxation dynamics of the intermediate scattering function at the structure factor maximum demonstrates that the relaxation dynamics changes from a high-temperature exponential decay to a stretched decay at around 1300 K. In addition, the structural relaxation time deviates from the hot-liquid Arrhenius behavior to a slower decay (see Fig. 3). These observations have been predicted as main signatures for the changes within the energy landscape^15,16^. For the simulations on a Lennard-Jones liquid it was reported that the minimum level for the atomic potential energy and hence the glasslike behavior is reached at a temperature $T=0.45$ of the high temperature plateau. It was noted that this temperature corresponds to the critical temperature $T_c$ from MCT. From the temperature evolution of the relaxation times we estimate that $T_c = 520~K$ for liquid aluminium (see Suppl. Information). From this value we can estimate the temperature where the free-diffusion-like movements set in as $T \approx 1150~K$ in reasonable good accordance with all here presented observations.

Some time ago the thermodynamics of aluminium has been studied over a wide temperature range^31^. If heterogenous melting can be avoided a crystal can be superheated. Eventually the entropy of the hot crystalline state will exceed the liquid one and the crystalline state becomes instable. Such a stability limit has been predicted for aluminium with a stability limit temperature $T_S = 1.38~\cdot T_m$. The stability limit can be regarded as the highest temperature for solid-like behavior in liquid aluminium, in remarkable good agreement with our observations. These thermodynamic investigations mirror perfectly the internal potential energy changes, when the landscape influenced regime is interpreted as the slowing down in the relaxation dynamics upon cooling associated to the enhancement of an icosahedral ordering^23,24^ that precedes either amorphisation or crystallisation in the undercooled region.

Previously we studied the generalized longitudinal viscosity $\eta _l(Q_0,\omega =0)$ and observed an increase upon cooling around $T \approx 1300~K$^30^, which might be the underlying origin for the slope change with decreasing temperature of the macroscopic shear viscosity. These changes in the viscosity might be related to the reported onset of cooperative particle movements as an origin for the increase in viscosity towards the glass transition^6^. The microscopic viscosity in a dense liquid is dominated by the forces between the particles and hence the change can be understood through the changing energy landscape. The relation of single particle dynamics with collective movements through the Stokes-Einstein relation has been investigated through a classical molecular dynamics simulation using a semi-empirical embedded atom model potential^19^. The authors concluded that about 180 K above their respective melting temperature of the simulated liquid aluminium the Stokes-Einstein relation is violated.

For liquid metals and alloys changes in the dynamics within the equilibrium liquid state have been reported. Violations of the Stokes-Einstein relation in experiment^37^ and MD-simulations have been reported^10^ and linked to the onset of correlated particle movements^12–14,38^. The diffusion coefficients for a wide range of glass forming liquids was investigated, including 11 alloys, and an Arrhenius crossover was reported at a temperature deep in the liquid state of alloys at about $2 \cdot T_g$^39^, a temperature range compatible with our estimate for liquid aluminium. All these reports from glass forming alloys demonstrate specific changes in the particle dynamics within the liquid state, separating a high-temperature liquid from the dense more viscous low-temperature liquid.

**Figure 5:**
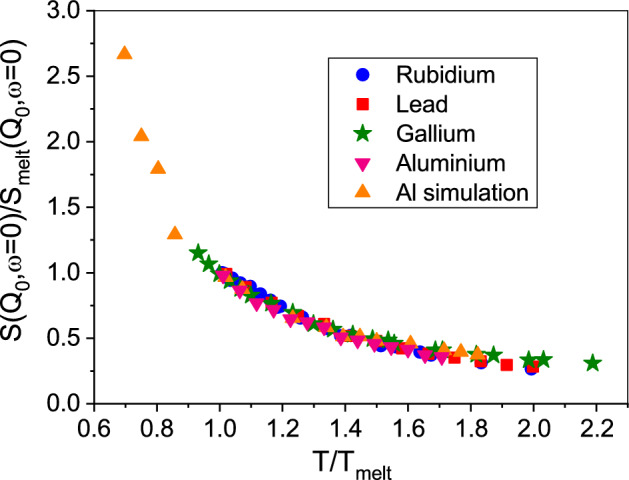
Amplitudes $S(Q,\omega =0)$, normalized against their melting temperature value, are depicted against a normalized temperature for different liquid metals. Data include rubidium^40^, lead^41^, gallium^42^ and the here presented experimental and simulation aluminium data.

These reports raise the question whether a transition in dynamics at a particular temperature range is a general feature of pure liquid metals. As a matter of fact, in Fig. 5 we plot the normalized amplitudes $S(Q_0,\omega =0)$ for liquid aluminium, gallium, lead and rubidium. The amplitudes are normalized to their respective value at the melting point and the temperature has also been normalized to their respective melting temperature $T_M$. Included are also the results from the present AIMD on liquid aluminium.

There is a remarkable agreement between all the reported amplitudes despite the very different interactions of these liquid metals. Because the amplitudes can be related to an average relaxation time, the relaxation dynamics in all these metals behave very similar with temperature and concomitant density change. We suggest that the observed crossover in dynamics in liquid metals is mirrored in changes of the inherent structure energy and evidences a temperature range above the melting point which signals the transition from a hot liquid to a more viscous liquid on its way to solidification. Whether this general feature extends to metallic alloys showing often a significant composition-dependent liquidus temperature remains to be investigated.

In summary, we presented experimental data on the structural relaxation of liquid aluminium in a combination with an extensive *ab initio* molecular dynamics simulation study over a wide temperature range, covering the liquid and supercooled state. We obtain a very good agreement between experimental and simulated data and find the hallmarks for the slowing down of structural relaxation dynamics at a temperature of about $1.4~T_M$. In the same temperature range we observe a decrease in the inherent structure energy, mirroring the changes in the dynamics. All observations suggest that the solidification process in liquid aluminium starts already around this temperature deep inside the equilibrium liquid state, and the similarity with other liquid metals points to an universal dynamic crossover in liquid dynamics. That temperature was also seen for pure Zr^43^. Given the generality of the potential energy landscape concept on which the observed phenomenon is based, it could extend to other metals and alloys, which remains to be investigated.

## Methods

The *ab initio* molecular dynamics simulations were mainly taken from Ref.^23^ and^44^ and extended here to the undercooled region. For the sake of self-consistency, we recall here the main technical details. The simulations of liquid Al were performed by means of the Vienna Ab initio Simulation Package (VASP)^45^. The local density approximation^46,47^ within projected augmented plane-waves^48^ was applied to all simulations with a plane-wave cutoff of 241 eV. Only the $\Gamma$ point is used to sample the Brillouin zone. $N = 256$ atoms are placed in a cubic simulation box of volume *V* with standard periodic boundary conditions. Newton’s equations of motion were solved numerically with Verlet’s algorithm in the velocity form with a time step of 1.5 fs, and phase-space trajectories were constructed within the canonical ensemble (*NVT*), by means of a Nosé thermostat to control the temperature *T*. We have shown that all these approximations reproduce the structural and transport properties of Al correctly^23,24^. The temperature evolution in the undercooled states was obtained by quenching the system stepwise down to 650 K with a temperature step of 50 K. For each temperature, the simulation cell was resized according to the experimental density^49^ and the run was continued for 30 ps before performing the next quench, resulting in an average cooling rate of $3.3 \cdot 10^{12}$ K/s. We mention that the calculated pressures for all the temperatures studied here were less than 1 GPa, so that we observe on average a constant pressure during the quenching. For temperatures ranging from $T = 1000$ K to 600 K, the run was continued for equilibration during a time up to 200 ps.

Among configurations produced after equilibration for the calculation of the properties, we have selected ten independent ones, regularly spaced in time, to extract their inherent structures^50^. To this end, an energy minimization procedure with the conjugate gradient method is imposed on each of these configurations at constant volume. it allows us to uncouple the vibrational motion from the underlying structural properties, since atoms are brought to a local minimum in the potential-energy surface for a given thermodynamic state^53^. For each the latter, ten independent configurations are taken out from the AIMD trajectory. In doing so, the minimized selected configurations explore several small local minima of the PEL basin for this temperature, with a Gaussian-like distribution and a standard deviation of the order of $10^{-3}$ eV. For one temperature, we have carried out this procedure with 50 configurations giving essentially the same result. For the single atom and collective dynamic properties, the self-intermediate and intermediate scattering functions, $F_s(Q,t)$ and *F*(*Q*, *t*), have been determined for all possible wave-vectors ${\mathbf {k}}$ compatible with the simulation box size.

Inelastic spectra on a levitated 2 mm diameter sphere of aluminium have been recorded at the ID28 beamline of the ESRF, Grenoble, France. Aerodynamic levitation is a simple way to suspend samples which can be heated independently with lasers. This technique was used successfully in neutron and synchrotron scattering experiments^51,52^. With the Si(12 12 12) reflection spectra at three temperatures between -30 and 30 meV have been recorded with an energy resolution of $FWHM=1.6~meV$ on eight analyser arms simultaneously. One analyser arm scanned at $Q=2.7$Å^-1^, which corresponds to the structure factor maximum of liquid aluminium. The temperature uncertainty, mainly reasoned by temperature drifts and uncertainties in the pyrometer calibration during the measurement, was about $\pm 30~K$. The intermediate scattering function *F*(*Q*, *t*) was obtained through a Fourier transform of the recorded spectra and a division through the Fourier transformed resolution function.

Neutron spectra of liquid aluminium in an alumina can have been measured at the IRIS-spectrometer of the ISIS Facility, UK. The spectrometer used an end energy of 7.38 meV, which provided an energy resolution of 0.055 meV (FWHM). This good energy resolution allowed a good separation of the liquid aluminium dynamics from the empty cell contribution and hence facilitates the necessary correction steps. Spectra for 14 temperatures between the melting point ($T_m = 933~K$) and 1593 K have been recorded with a dynamic range between -3 to 4.5 meV. The empty can contribution has been subtracted, an absolute calibration performed and the spectra in constant *Q* and energy bins divided. More experimental details can be found in^28^.

## Supplementary information

Supplementary Information 1.
