# Supplementary material for: The intimate relationship between structural relaxation and the energy landscape of monatomic liquid metals
Source: Sci Rep. 2021 Jun 3;11:11815. doi: 10.1038/s41598-021-91062-0 (PMC8175717; doi:10.1038/s41598-021-91062-0)
Supplement: Supplementary file 1 — Supplementary Information 1. [file 41598_2021_91062_MOESM1_ESM.pdf]

The intimate relationship between structural relaxation and the energy landscape of monatomic liquid metals.

Franz Demmel
ISIS Facility, Rutherford Appleton Laboratory, Didcot, OX11 0QX, United Kingdom

Louis Hennet
ICMN, CNRS and University of Orleans, 45071 Orléans, France

Noel Jakse
Univ. Grenoble Alpes, CNRS, Grenoble INP, SIMaP, F-38000 Grenoble, France
(Dated: March 11, 2021)

SUPPORTING INFORMATION

Experimental spectra

In Fig. 1 we plot a neutron spectrum and an x-ray spectrum taken near the melting point. The spectra have been peak normalized and demonstrate a good agreement. The large dynamic range covered by the x-ray experiment allows to access small time steps in the Fourier transformed $F(Q,t)$ spectra, whereas $F(Q,t)$ data from the neutron spectra have only limited value to study the relaxation dynamics in time. Otherwise is the broadening due to the energy resolution non-negligible in the case of inelastic x-ray scattering. In Fig. 2 we present a comparison between neutron data and x-ray data in the time domain. Displayed are F(Q,t) spectra, obtained through Fourier transformation and normalized to their $t = 0$ value, which is $S(Q)$. It demonstrates the limitation of time steps from the energy range limited neutron spectra. Obviously the analysis of F(Q,t) spectra from neutron data would rely at higher temperatures only on a few time steps. Hence the here presented neutron spectra have been analysed in the energy domain. However, despite the very different experimental approaches the resulting intermediate scattering functions agree perfectly.

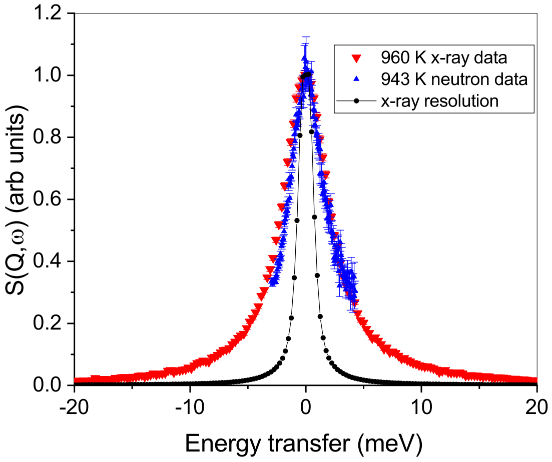

FIG. 1. A neutron and a x-ray spectrum is displayed. Included is the energy resolution of the x-ray spectrometer.

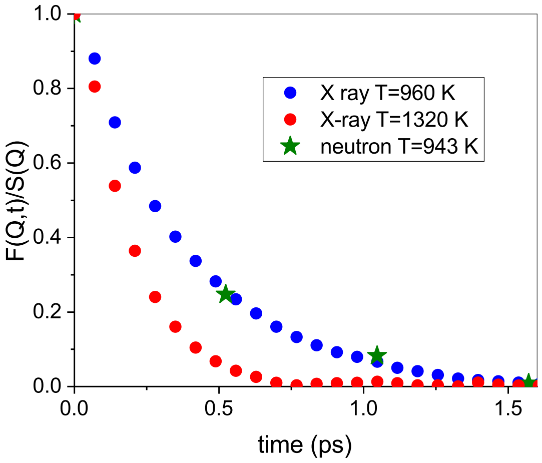

FIG. 2. $F(Q,t)$ spectra are shown from neutron and a x-ray scattering.

Average relaxation time

The amplitude $S(Q_0, \omega = 0)$ is related to an integral of the intermediate scattering function $F(Q, t)$:

$$S(Q, \omega = 0) = \frac{1}{2\pi} \int_{-\infty}^{\infty} F(Q, t) exp(-i\omega t) dt \mid_{\omega=0} = \frac{1}{\pi} \int_{0}^{\infty} F(Q, t) dt \quad (1)$$

The integral is the area under the relaxation curve and can be used as a definition for an average relaxation time. An estimate for an average relaxation time τ_{ave} of $F(Q, t)$ can be obtained through [1]:

$$\tau_{ave} = \int_0^{\infty} dt \frac{< n^*(Q, t), n(Q, 0) >}{< n^*(Q, 0), n(Q, 0) >} = \frac{\int_0^{\infty} dt F(Q, t)}{S(Q)} = \frac{\pi S(Q_0, \omega = 0)}{S(Q_0)} \quad (2)$$

As input the temperature dependent structure factor maximum $S(Q_0)$ is needed. We took the simulated structure factor maximum data and interpolated to the respective experimental temperatures. Fig. 3 shows the $S(Q_0)$ values

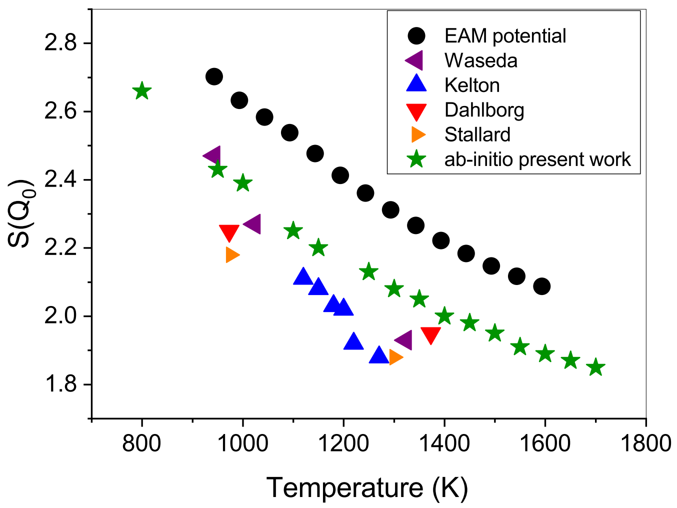

FIG. 3. Peak values $S(Q_0)$ of liquid aluminium are plotted from experiment, EAM simulation [6] and from the present ab-initio simulation. X-ray data are from *Waseda* [2] and from *Kelton* [4]. Neutron scattering data are *Dahlborg* from [5] and *Stallard* from [3].

from AIMD in comparison with experimental data from the literature and data obtained with a classical embedded atom model (EAM) potential. The AIMD data seem to agree better with the available experimental data points, whereas the EAM potential appear to over emphasize structural ordering in the liquid.

Then average relaxation times τ_{ave} have been calculated and are plotted in Fig. 4 on a logarithmic scale against the inverse temperature. These values are compared with fits of the simulated $F(Q_0, t)$ data, where the fit of a stretched exponential function was applied to the whole time scale in order to obtain an estimate for a comparable average relaxation time. There is an overall and surprising good agreement between the two completely different methods. Note that the experimental average relaxation times are strongly influenced by the temperature dependence of the structure factor $S(Q_0)$, which is obtained through a different method. Applying a linear fit to the high temperature data points (larger than $T \approx 1300\ K$) the extrapolated line indicates a deviation toward slower relaxation times with decreasing temperature.

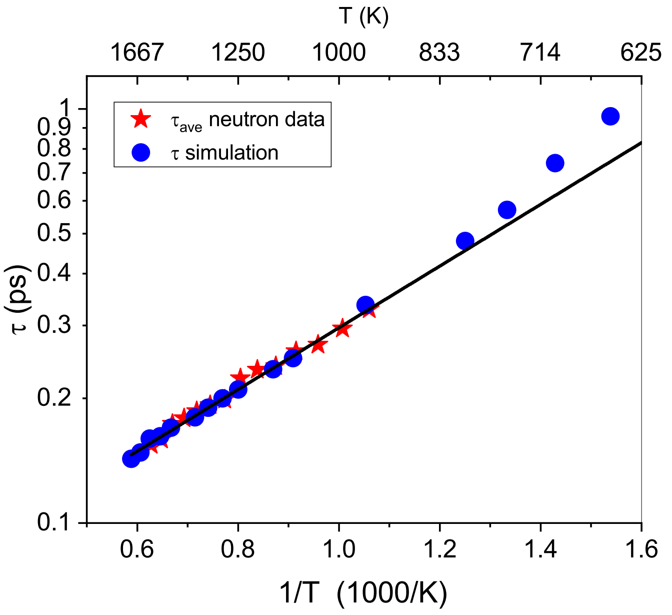

FIG. 4. Average relaxation times τ_{ave} (stars) obtained from the neutron scattering amplitudes $S(Q_0, \omega = 0)$ are plotted on a logarithmic scale against the inverse temperature. Included are (circles) fitted average relaxation times from the simulated $F(Q_0, t)$ data. The line marks a high-temperature liquid extrapolation to lower temperatures.

MCT power-law fit

Mode coupling theory predicts a divergence of the structural relaxation time and consequently other related quantities like the diffusion coefficient at a critical temperature T_c [7]. A power-law behavior near T_c is expected:

$$\tau = \tau_0 (T - T_c)^{-\gamma} \tag{3}$$

with an exponent γ which should be universal for all divergent properties. Not exact known is the range of temperatures above T_c where these relations are valid. In Fig. 5 we plot the relaxation times from the AIMD intermediate scattering functions. We fitted a power-law to the data points below 1400 K and obtained a $T_c = 520\ K$ with a $\gamma = 1.05$. Previously the experimentally determined linewidth was extrapolated linearly to zero width to estimate when liquid aluminium comes to an arrest [6]. That extrapolation delivered $T \approx 600\ K$ as an estimate for the arrest of liquid aluminium, not too far from the above obtained T_c.

Excess entropy

The structural indicator can be advantageously revealed through the excess entropy S_{ex} [8, 9]. As a matter of fact, it can be written reliably in its approximate two-body form as

$$S_{ex} \simeq S_2 = -2\pi\rho \int (g(r) \ln g(r) - (g(r) - 1))\, dr, \tag{4}$$

which depends on the pair-correlation function $g(r)$ and the number density ρ. The quantity $\exp(-S_{ex})$, which is inversely proportional to the number of accessible states, is shown in Fig. 5. It clearly shows a crossover at $1.4T_M$ as shown in Fig. 2 of the main text on $S(Q, \omega = 0)$ and indicate the mechanisms of slowing down of the dynamics.

[1] Berne, B.J. and Pecora, R. *Dynamic Light Scattering* Dover Publications, New York p15 (2000)

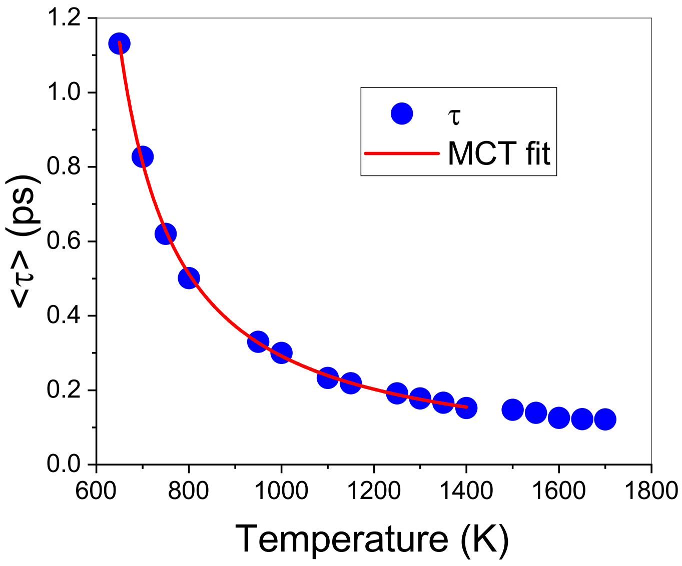

FIG. 5. The relaxation times $< \tau >$ (circles) obtained from the AIMD are plotted against the temperature. Included as a line is the fit with a MCT power-law prediction.

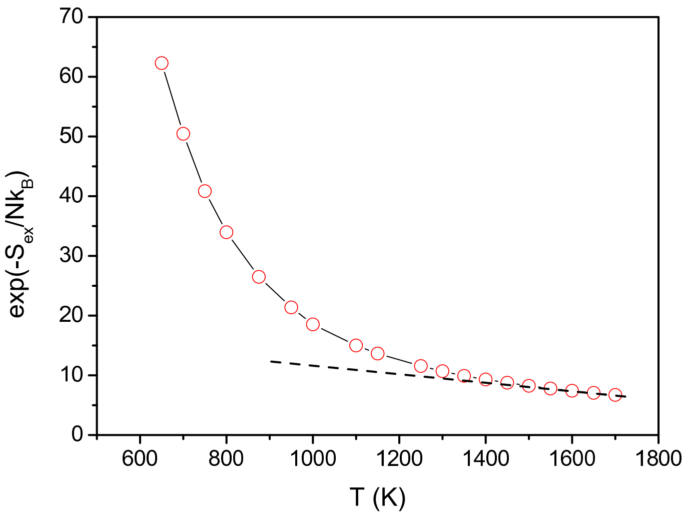

FIG. 6. The excess entropy as a function of temperature from *ab initio* molecular dynamics simulations. Included as a line is a fit through the high temperature points.

[2] Waseda, Y. *The Structure of Non-Crystalline Materials* McGraw Hill, New York (1980)
[3] Stallard, J.M. and Davis, C.M. Liquid-Aluminium structure factor by neutron diffraction. Phys. Rev. A **8** 368 (1973).
[4] Mauro, N.A., Bendert, J.C., Vogt, A.J., Gewin, J.M. and Kelton, K.F. High energy x-ray scattering studies of the local order in liquid Al. J. Chem. Phys. **135** 044502 (2011).
[5] Dahlborg, U., Kramer, M.J., Besser, M., Morris, J.C., Calvo-Dahlbourg, M. Structure of molten Al and eutectic Al?Si alloy studied by neutron diffraction, J. Non-Cryst. Solids **361**, 63 (2013).
[6] Demmel, F., Fraile, A., Szubrin, D., Pilgrim, W.C. and Morkel, C. Experimental evidence for a dynamical crossover in liquid aluminium. J.Phys.: Condens. Matter **27** 455102 (2015).
[7] Kob, W. Supercooled Liquids, the glass transition, and computer simulations. Les Houches Summer School - Session LXXVII (2002).
[8] Rosenfeld, Y. Relation between the transport coefficients and the internal entropy of simple systems, Phys. Rev. A **15**, 2545 (1977).
[9] Baranyai, A., Evans, D. J. Direct entropy calculation from computer simulation of liquids, Phy. Rev. A **40**, 3817 (1989).
